# Crystal structure of tetra­kis­(μ-4-benzyl-4*H*-1,2,4-triazole-κ^2^ *N* ^1^:*N* ^2^)tetra­fluoridodi-μ_2_-oxido-dioxidodisilver(I)divanadium(V)

**DOI:** 10.1107/S2056989022001712

**Published:** 2022-03-15

**Authors:** Ganna A. Senchyk, Andrey B. Lysenko, Eduard B. Rusanov, Kostiantyn V. Domasevitch

**Affiliations:** a Inorganic Chemistry Department, Taras Shevchenko National University of Kyiv, Volodymyrska Street, 64, Kyiv 01033, Ukraine; b Institute of Organic Chemistry, Murmanska Street, 5, Kyiv, 02660, Ukraine

**Keywords:** silver(I), vanadium(V) oxofluoride, 1,2,4-triazole, Hirshfeld surface analysis, crystal structure

## Abstract

The crystal structure of the title compound, [Ag_2_(VO_2_F_2_)_2_(C_9_H_9_N_3_)_4_], is presented. The mol­ecular complex is based on the heterobimetallic Ag^I^—V^V^ fragment {Ag^I^_2_(V^V^O_2_F_2_)_2_(*tr*)_4_} supported by four 1,2,4-triazole ligands [4-benzyl-(4*H*-1,2,4-triazol-4-yl)]. The triazole functional group demonstrates homo- and heterometallic connectivity (Ag—Ag and Ag—V) of the metal centers through the [–NN–] double and single bridges, respectively. The vanadium atom possesses a distorted trigonal–bipyramidal coordination environment [VO_2_F_2_N] with the Reedijk structural parameter τ = 0.59. In the crystal, C—H⋯O and C—H⋯F hydrogen bonds as well as C—H⋯*π* contacts are observed involving the organic ligands and the vanadium oxofluoride anions. A Hirshfeld surface analysis of the hydrogen-bonding inter­actions is also described.

## Abstract 2

The title heterobimetallic silver(I)–vanadium(V) oxide-fluoride compound is built on the {Ag_2_(VO_2_F_2_)_2_(*tr*)_4_} secondary building unit supported by 1,2,4-triazole ligands [4-benzyl-(4*H*-1,2,4-triazol-4-yl)].

## Chemical context

There is considerable inter­est in the chemistry of organic–inorganic hybrids, including the vanadium oxide–fluoride (VOF) matrix, which is motivated by the numerous potential applications in catalysis, magnetism, optics, *etc.* (Dolbecq *et al.*, 2010 ▸; Monakhov *et al.*, 2015 ▸). Incorporation of silver(I) in VOF solid can afford materials such as Ag_4_V_2_O_6_F_2_ (Sorensen *et al.*, 2005 ▸; Albrecht *et al.*, 2009 ▸) and Ag_3_VO_2_F_4_ (Chamberlain *et al.*, 2010 ▸), which are attractive candidates for solid-state battery technologies. The formation of Ag^I^–VOF heterobimetallic secondary building units (SBUs) in coordination compounds remains a non-trivial challenge. The 1,2,4-triazole heterocycle, as a functional group, demonstrates a favorable coordination affinity towards Ag^I^ cations, connecting them into polynuclear units (Aromí *et al.*, 2011 ▸). At the same time, it possesses a hidden capability to bind two different metal ions through a short –NN– bridge, usually Cu^II^–*tr*–Mo^VI^ (Tian *et al.*, 2011 ▸; Lysenko *et al.*, 2016 ▸; Senchyk *et al.*, 2017 ▸; Zhu *et al.*, 2012 ▸) but there are some other rare examples including Cu^I^–*tr*–V^IV^ (Sharga *et al.*, 2010 ▸) and Ag^I^–*tr*–Mo^VI^ (Tian *et al.*, 2017 ▸). This may be realized in the case of constructing SBUs with a terminal *N* ^1^-triazole function that has an open site accessible to coordination. We demonstrated this principle in the self-association of Ag^I^–VOF heterobimetallic coordination compounds based on {Ag^I^_2_(V^V^O_2_F_2_)_2_(*tr*)_4_} SBUs with bi-1,2,4-triazole ligands with different geometries (Senchyk *et al.*, 2012 ▸). Such units seem to be very favorable and stable, and form even in the presence of a heterobifunctional 1,2,4-triazole-carboxyl­ate ligand (Senchyk *et al.*, 2019 ▸). In the present contribution we extend the library of Ag^I^–VOF compounds, adding the title complex [Ag_2_(VO_2_F_2_)_2_(*tr-CH_2_Ph*)_4_] (**I**), which has the ligand 4-benzyl-(4*H*-1,2,4-triazol-4-yl) (*tr*-CH_2_Ph). 
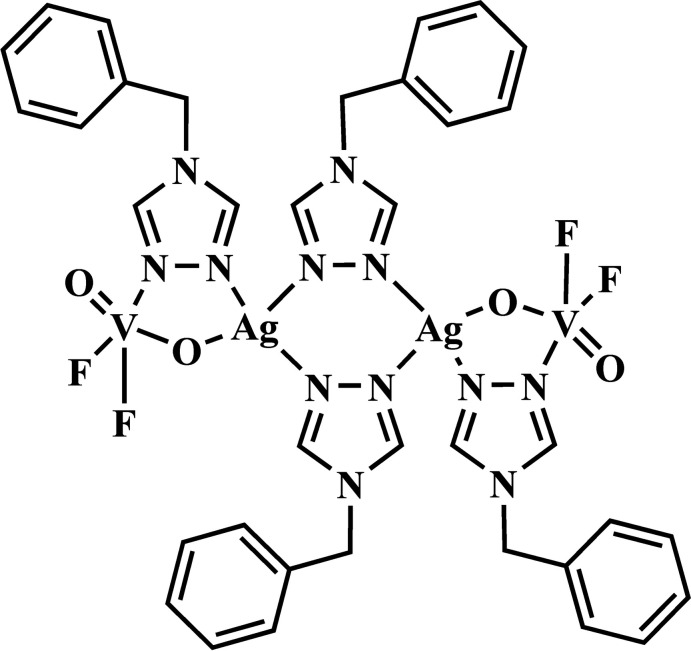


## Structural commentary

**Figure 1:**
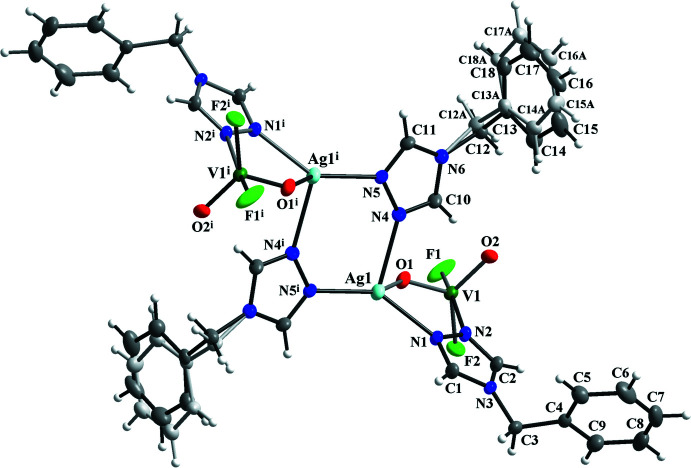
The mol­ecular structure of compound **I**, showing the atom-labeling scheme [symmetry code: (i) −*x*, −*y* + 1, −*z*]. Displacement ellipsoids are drawn at the 30% probability level.

Compound **I** crystallizes in the monoclinic space group *P*2_1_/*c*. Its asymmetric unit contains one Ag^I^ cation, one [V^V^O_2_F_2_]^−^ anion and two organic ligands (*tr*-CH_2_Ph), which, after inversion across a center of symmetry, form the mol­ecular tetra­nuclear cluster {Ag^I^_2_(V^V^O_2_F_2_)_2_(*tr-*CH_2_Ph)_4_} (Fig. 1 ▸). Two 1,2,4-triazole ligands bridge two adjacent silver atoms [the Ag⋯Ag^i^ distance is 4.2497 (5) Å; symmetry code (i) −*x*, −*y* + 1, −*z*], while the other two link Ag and V centers [the Ag⋯V distance is 3.8044 (6) Å]. Thus, the coordination environment of the Ag^I^ cation can be described as [AgN_3_O] with typical Ag—N(triazole) bond lengths [in the range of 2.197 (2) – 2.390 (3) Å] and a slightly elongated Ag—O bond [2.562 (2) Å] (Table 1 ▸). The V^V^ atom possesses a distorted trigonal–bipyramidal coordination environment [VO_2_F_2_N] with V—F [1.828 (2) and 1.8330 (18) Å], two short V—O [1.632 (2) and 1.660 (2) Å] and elongated V—N [2.203 (2) Å] bonds (Table 1 ▸). The geometry of the vanadium oxofluoride polyhedra is characterized by the Reedijk structural parameter τ (Addison *et al.*, 1984 ▸) of 0.59 (for a square-pyramidal geometry, τ = 0 and for trigonal–bipyramidal, τ = 1). A bond-valence-sum calculation for the {VO_2_F_2_N} polyhedra confirms the +5 oxidation state for the vanadium atom.

## Supra­molecular features

**Figure 2:**
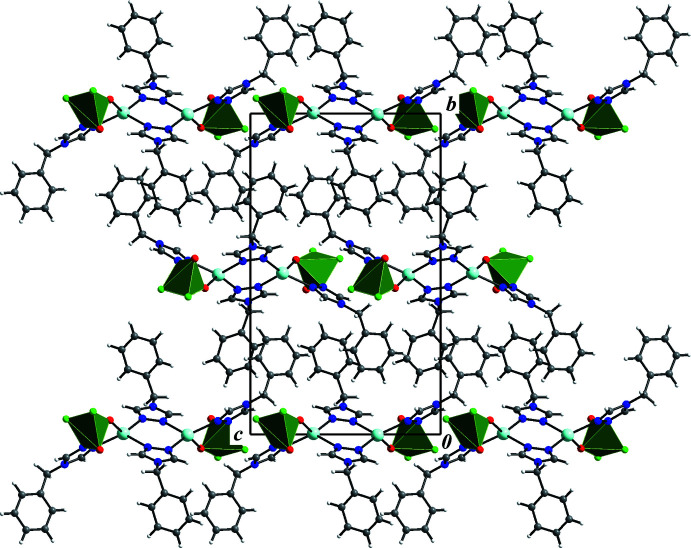
Projection on the *bc* plane showing the crystal packing of compound **I**. Vanadium oxofluoride anions are shown as polyhedra. [Atoms are colored as follows: silver – cyan, vanadium – dark green, oxygen – red, fluorine – green, nitro­gen – blue, carbon – gray, hydrogen – white.]

Since the organic ligand contains a hydro­phobic benzyl tail, the crystal structure of **I** involves no solvate water mol­ecules. Thus, the only hydrogen bonds observed are of the type C—H⋯O, C—H⋯F and C—H⋯*π* contacts (Figs. 2 ▸ and 3 ▸, Table 2 ▸). The central 1,2,4-triazole unit, which bridges two Ag ions, displays intra­molecular C10—H10⋯O2 [3.082 (4) Å] and inter­molecular C11—H11⋯F1^v^ [2.935 (4) Å, symmetry code (v) −*x* + 1, −*y* + 1, −*z*] hydrogen-bond contacts. The other triazole group, which provides the heterometallic Ag–V linkage, forms bifurcated C—H⋯O and C—H⋯F contacts with vanadium oxofluoride anions of neighboring mol­ecular complexes. Additionally, methyl­ene –CH_2_– fragments show directed C—H⋯O and C—H⋯F contacts to the VOF fragments. The phenyl rings are here oriented towards each other in an edge-to-face C—H⋯*π* inter­action mode.

**Figure 4:**
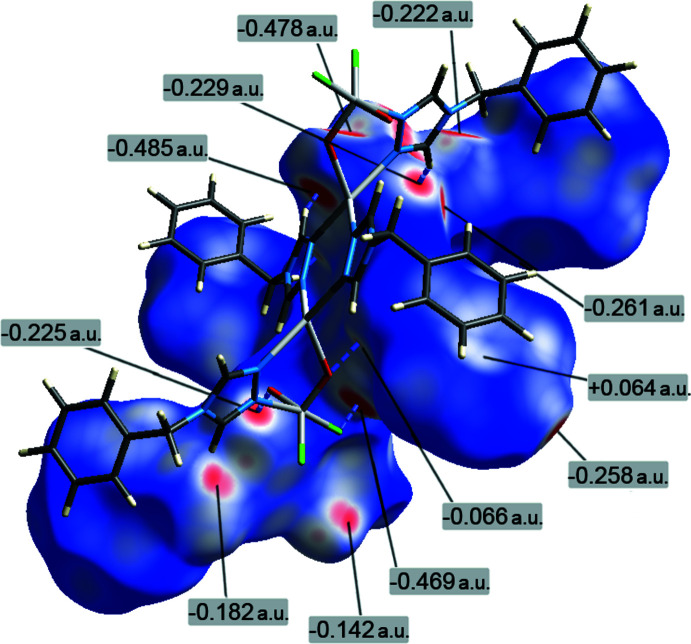
The Hirshfeld surface of the title compound mapped over *d*_norm_ in the color range −0.488 (red) to 1.385 (blue) a.u., in the environment of the closest neighbor [symmetry code: −*x* + 1, −*y* + 1, −*z*], with the red spots indicating different kinds of inter­molecular inter­actions.

Supra­molecular inter­actions in the title structure were studied through Hirshfeld surface analysis (Spackman & Byrom, 1997 ▸; McKinnon *et al.*, 2004 ▸; Hirshfeld, 1977 ▸; Spackman & McKinnon, 2002 ▸), performed with *CrystalExplorer17* (Turner *et al.*, 2017 ▸), taking into account only the major contribution of the disordered group. The Hirshfeld surface, mapped over *d*_norm_ using a fixed color scale of −0.488 (red) to 1.385 (blue) a.u. visualizes the set of shortest inter­molecular contacts (Fig. 4 ▸). All of them correspond to the hydrogen-bond inter­actions, which fall into three categories. The strongest hydrogen bonds to F-atom acceptors are reflected by the most prominent red spots (−0.469 to −0.488 a.u.), whereas a group of medium intensity spots (−0.182 to −0.261 a.u.) identify weaker C—H⋯O bonds with the terminal oxide O2. However, even more distal inter­actions with the bridging oxide O1 are still distinguishable on the surface, in the form of very diffuse, less intense spots (−0.066 to −0.142 a.u.).

**Figure 5:**
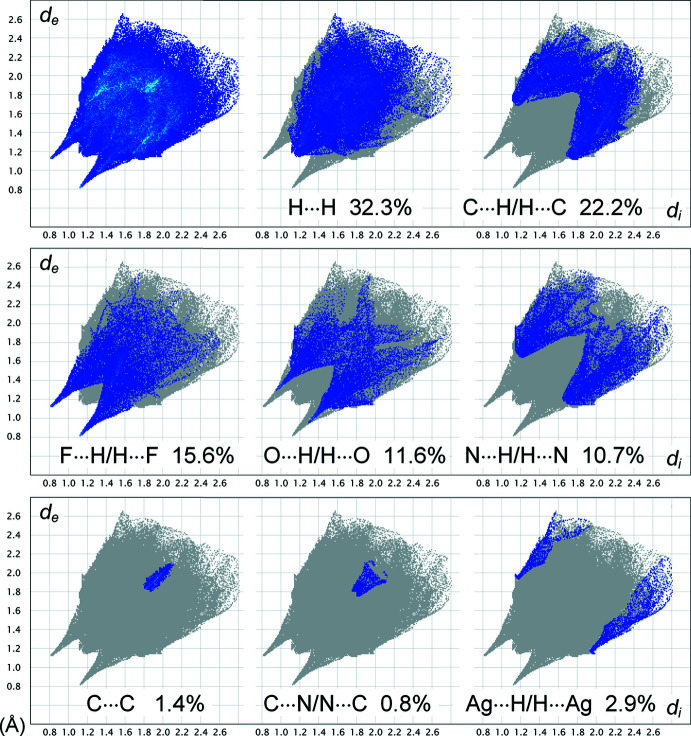
Two-dimensional fingerprint plots for the title compound, and those delineated into the principal contributions of H⋯H, C⋯H/H⋯C, F⋯H/H⋯F, O⋯H/H⋯O, N⋯H/H⋯N, C⋯C, C⋯N/N⋯C and Ag⋯H/H⋯Ag contacts. Other observed contacts are N⋯N (0.4%), C⋯F/F⋯C (0.1%) and C⋯O/O⋯C (0.1%).

The contribution of different kinds of inter­atomic contacts to the Hirshfeld surface is shown in the fingerprint plots in Fig. 5 ▸. A significant fraction of the *E*⋯H/H⋯*E* (*E* = C, N, O, F) contacts (in total 60.1%) suggests the dominant role of the hydrogen-bond inter­actions. The strongest ones (*E* = O, F) have a similar nature and they are reflected by pairs of spikes pointing to the lower left of the plot. However, the contribution from the contacts with F-atom acceptors is higher (15.6% for F⋯H/H⋯F and 11.6% for O⋯H/H⋯O) and they are also essentially shorter, as indicated by different lengths of the spikes (the shortest contacts are F⋯H = 2.0 and O⋯H = 2.2 Å). One may suppose that the preferable sites for hydrogen bonding of the vanadium oxofluoride groups are the F atoms. This is consistent with the results of Hirshfeld analysis for the [VOF_5_]^2−^ anion 4,4′-(propane-1,3-di­yl)bis­(4*H*-1,2,4-triazol-1-ium) salt (Senchyk *et al.*, 2020 ▸).

The plots indicate close resemblance of the N⋯H/H⋯N (10.7%) and C⋯H/H⋯C (22.2%) contacts, which appear as pairs of nearly identical, very diffuse and short features (N⋯H = 2.9 and C⋯H = 2.9 Å). Both of them correspond to edge-to-face stacking or C—H⋯*π* inter­actions involving either the phenyl or triazole rings. The contribution from mutual *π–π* inter­actions of the latter delivers minor fractions of the C⋯C, N⋯N and C⋯N/N⋯C contacts, which account in total for only 2.6%. The shortest contact of this series [C⋯N = 3.5 Å] exceeds the sum of the van der Waals radii [3.25 Å] and *π–π* inter­actions are not associated with red spots of the *d*_norm_ surface. A comparable contribution is due to the distal anagostic contacts Ag⋯H/H⋯Ag (2.9%) with the polarized methyl­ene H atoms. There are no mutual *π–π* inter­actions involving phenyl rings, which are responsible for larger fractions of the C⋯C contacts in the case of polycyclic species (Spackman & McKinnon, 2002 ▸).

## Database survey

A structure survey was carried out in the Cambridge Structural Database (CSD version 5.43, update of November 2021; Groom *et al.*, 2016 ▸) for 4-benzyl-(4*H*-1,2,4-triazol-4-yl) and it revealed five hits for coordination compounds based on this ligand. There are no examples of Ag^I^ compounds, only two Fe^II^ complexes [FAYQAA (Pittala *et al.*, 2017*a* ▸) and XASVEV (Pittala *et al.*, 2017*b* ▸)] and three Cu^II^–POM complexes [YUGLIX and YUGLOD (Tian *et al.*, 2015 ▸) and ZUXLAI (Zhang *et al.*, 2020 ▸)]. Moreover, there are no examples of heterometallic connection through an –NN– triazole bridge for the 4-benzyl-(4*H*-1,2,4-triazol-4-yl) ligand.

## Synthesis and crystallization

4-Benzyl-(4*H*-1,2,4-triazol-4-yl) (*tr*-CH_2_Ph) was synthesized by refluxing benzyl­amine (5.35 g, 50.0 mmol) and di­methyl­formamide azine (17.75 g, 125.0 mmol) in the presence of toluene­sulfonic acid monohydrate (0.86 g, 5.0 mmol) as a catalyst in DMF (30.0 ml).

Compound **I** was prepared under hydro­thermal conditions. A mixture of AgOAc (16.7 mg, 0.100 mmol), *tr*-CH_2_Ph (20.7 mg, 0.130 mmol), V_2_O_5_ (9.1 mg, 0.050 mmol) and 5 mL of water with aqueous HF (50%, 150 µL, 4.33 mmol) was added into a Teflon vessel. Then the components were heated at 423 K for 24 h and slowly cooled to room temperature over 50 h, yielding light-yellow prisms of **I** (yield 33.4 mg, 61%).

## Refinement

Crystal data, data collection and structure refinement details are summarized in Table 3 ▸. For one of the organic ligands, the benzyl linkage (C12–C18) is unequally disordered over two overlapping positions with refined partial contribution factors of 0.68 (3) and 0.32 (3). The major part of the disorder was freely refined anisotropically, while atoms of the minor contributor were refined anisotropically with a restrained geometry for the phenyl ring, rigid-bond restraints applied to the –CH_2_C_6_H_5_ linkage and similarity restraints applied to the closely separated contributions of C12 and C12*A*, C13 and C13*A*. H atoms were positioned geometrically and refined as riding, with C—H = 0.93 Å (CH) and 0.97 Å (CH_2_) and with *U*_iso_(H) = 1.2*U*_eq_(C).

## Supplementary Material

Crystal structure: contains datablock(s) I. DOI: 10.1107/S2056989022001712/dj2039sup1.cif

Structure factors: contains datablock(s) I. DOI: 10.1107/S2056989022001712/dj2039Isup2.hkl

CCDC reference: 2151864

Additional supporting information:  crystallographic information; 3D view; checkCIF report

## Figures and Tables

**Figure 3:**
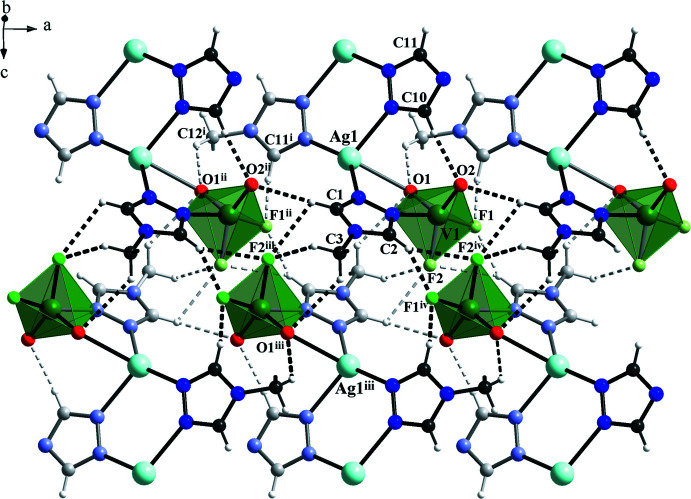
Hydrogen-bonding arrangement in the structure of **I** showing C—H⋯O and C—H⋯F contacts [symmetry codes: (ii) *x* − 1, *y*, *z*; (iii) −*x*, −*y* + 1, −*z* + 1; (iv) −*x* + 1, −*y* + 1, −*z* + 1; (v) −*x* + 1, −*y* + 1, −*z*; (vi) *x*, −*y* + 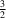 , *z* − 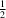 .]. Phenyl groups are omitted for clarity.

**Table 1:** Selected geometric parameters (Å, °)

Ag1—N5^i^	2.197 (2)	V1—O2	1.660 (2)
Ag1—N1	2.233 (2)	V1—F1	1.828 (2)
Ag1—N4	2.390 (3)	V1—F2	1.8330 (18)
Ag1—O1	2.562 (2)	V1—N2	2.203 (2)
V1—O1	1.632 (2)		
			
N5^i^—Ag1—N1	140.62 (9)	O1—V1—F2	117.63 (10)
N5^i^—Ag1—N4	102.45 (9)	O2—V1—F2	132.25 (10)
N1—Ag1—N4	112.90 (9)	F1—V1—F2	86.76 (10)
N5^i^—Ag1—O1	129.87 (8)	O1—V1—N2	87.14 (10)
N1—Ag1—O1	75.28 (8)	O2—V1—N2	88.78 (11)
N4—Ag1—O1	79.39 (8)	F1—V1—N2	167.32 (10)
O1—V1—O2	108.04 (11)	F2—V1—N2	80.59 (9)
O1—V1—F1	99.57 (11)	V1—O1—Ag1	128.89 (11)
O2—V1—F1	99.21 (13)		

Symmetry code: (i) 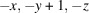 .

**Table 2:** Hydrogen-bond geometry (Å, °)

*D*—H⋯*A*	*D*—H	H⋯*A*	*D*⋯*A*	*D*—H⋯*A*
C1—H1⋯O2^ii^	0.93	2.44	3.289 (4)	153
C1—H1⋯F2^iii^	0.93	2.63	3.108 (4)	113
C2—H2⋯F1^iv^	0.93	2.07	2.935 (4)	154
C2—H2⋯F2^iv^	0.93	2.60	3.304 (4)	133
C3—H3*A*⋯O1^iii^	0.97	2.73	3.465 (4)	133
C3—H3*B*⋯F2^iii^	0.97	2.37	3.006 (4)	123
C10—H10⋯O2	0.93	2.16	3.082 (4)	170
C11—H11⋯F1^v^	0.93	2.07	2.935 (4)	153
C12—H12*A*⋯O1^v^	0.97	2.65	3.388 (2)	133
C16—H16⋯O2^vi^	0.93	2.42	3.339 (9)	172
C18—H18⋯O1^v^	0.93	2.83	3.589 (15)	139

Symmetry codes: (ii) 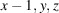 ; (iii) 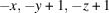 ; (iv) 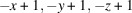 ; (v) 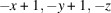 ; (vi) 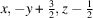 .

**Table 3:** Experimental details

Crystal data	
Chemical formula	[Ag_2_V_2_F_4_O_4_(C_9_H_9_N_3_)_4_]
*M*_r_	1094.39
Crystal system, space group	Monoclinic, *P*2_1_/*c*
Temperature (K)	296
*a*, *b*, *c* (Å)	7.5484 (2), 21.2439 (6), 12.5910 (4)
β (°)	90.910 (2)
*V* (Å^3^)	2018.81 (10)
*Z*	2
Radiation type	Mo *K*α
μ (mm^−1^)	1.48
Crystal size (mm)	0.27 × 0.14 × 0.12

Data collection	
Diffractometer	Bruker APEXII area-detector
Absorption correction	multi-scan (*SADABS*; Bruker, 2008 ▸)
*T*_min_, *T*_max_	0.657, 0.856
No. of measured, independent and observed [*I* > 2σ(*I*)] reflections	22923, 5125, 3468
*R*_int_	0.044
(sin θ/λ)_max_ (Å^−1^)	0.676

Refinement	
*R*[*F* ^2^ > 2σ(*F* ^2^)], *wR*(*F* ^2^), *S*	0.038, 0.078, 1.02
No. of reflections	5125
No. of parameters	323
No. of restraints	65
H-atom treatment	H-atom parameters constrained
Δρ_max_, Δρ_min_ (e Å^−3^)	0.58, −0.42

Computer programs: *APEX2* and *SAINT* (Bruker, 2008 ▸), *SHELXS97* (Sheldrick, 2008 ▸), *SHELXL2014/7* (Sheldrick, 2015 ▸), *DIAMOND* (Brandenburg, 1999 ▸) and *WinGX* (Farrugia, 2012 ▸).

## supplementary crystallographic information

### Crystal data

**Table:**

[Ag_2_V_2_F_4_O_4_(C_9_H_9_N_3_)_4_]	*F*(000) = 1088
*M**_r_* = 1094.39	*D*_x_ = 1.800 Mg m^−^^3^
Monoclinic, *P*2_1_/*c*	Mo *K*α radiation, λ = 0.71073 Å
*a* = 7.5484 (2) Å	Cell parameters from 4931 reflections
*b* = 21.2439 (6) Å	θ = 2.5–23.8°
*c* = 12.5910 (4) Å	µ = 1.48 mm^−^^1^
β = 90.910 (2)°	*T* = 296 K
*V* = 2018.81 (10) Å^3^	Block, colorless
*Z* = 2	0.27 × 0.14 × 0.12 mm

### Data collection

**Table:**

Bruker APEXII area-detector diffractometer	3468 reflections with *I* > 2σ(*I*)
Radiation source: fine-focus sealed tube	*R*_int_ = 0.044
ω scans	θ_max_ = 28.7°, θ_min_ = 1.9°
Absorption correction: multi-scan (SADABS; Bruker, 2008)	*h* = −10→10
*T*_min_ = 0.657, *T*_max_ = 0.856	*k* = −26→28
22923 measured reflections	*l* = −16→14
5125 independent reflections	

### Refinement

**Table:**

Refinement on *F*^2^	Primary atom site location: structure-invariant direct methods
Least-squares matrix: full	Secondary atom site location: difference Fourier map
*R*[*F*^2^ > 2σ(*F*^2^)] = 0.038	Hydrogen site location: inferred from neighbouring sites
*wR*(*F*^2^) = 0.078	H-atom parameters constrained
*S* = 1.02	*w* = 1/[σ^2^(*F*_o_^2^) + (0.0194*P*)^2^ + 2.1764*P*] where *P* = (*F*_o_^2^ + 2*F*_c_^2^)/3
5125 reflections	(Δ/σ)_max_ = 0.001
323 parameters	Δρ_max_ = 0.58 e Å^−^^3^
65 restraints	Δρ_min_ = −0.42 e Å^−^^3^

### Special details

**Table:**

Geometry. All esds (except the esd in the dihedral angle between two l.s. planes) are estimated using the full covariance matrix. The cell esds are taken into account individually in the estimation of esds in distances, angles and torsion angles; correlations between esds in cell parameters are only used when they are defined by crystal symmetry. An approximate (isotropic) treatment of cell esds is used for estimating esds involving l.s. planes.

### Fractional atomic coordinates and isotropic or equivalent isotropic displacement parameters (Å^2^)

**Table:**

	*x*	*y*	*z*	*U*_iso_*/*U*_eq_	Occ. (<1)
Ag1	0.00892 (3)	0.49751 (2)	0.16871 (2)	0.04174 (9)	
V1	0.43103 (7)	0.48341 (2)	0.33615 (4)	0.03165 (13)	
F1	0.6173 (3)	0.42871 (13)	0.32820 (16)	0.0802 (8)	
F2	0.3957 (2)	0.45217 (9)	0.46985 (15)	0.0471 (5)	
O1	0.3065 (3)	0.45593 (10)	0.23966 (17)	0.0407 (5)	
O2	0.5296 (3)	0.54823 (11)	0.29314 (16)	0.0424 (6)	
N1	0.0417 (3)	0.54163 (12)	0.32878 (19)	0.0315 (6)	
N2	0.2025 (3)	0.54132 (11)	0.38181 (19)	0.0301 (6)	
N3	0.0147 (3)	0.59315 (11)	0.47783 (18)	0.0292 (6)	
N4	0.2055 (4)	0.54025 (13)	0.0405 (2)	0.0386 (6)	
N5	0.1929 (3)	0.54905 (12)	−0.06853 (19)	0.0345 (6)	
N6	0.4464 (3)	0.58619 (12)	−0.0125 (2)	0.0342 (6)	
C1	−0.0684 (4)	0.57241 (14)	0.3893 (2)	0.0337 (7)	
H1	−0.1876	0.5790	0.3732	0.040*	
C2	0.1821 (4)	0.57219 (14)	0.4702 (2)	0.0328 (7)	
H2	0.2710	0.5788	0.5209	0.039*	
C3	−0.0650 (4)	0.62351 (15)	0.5708 (2)	0.0400 (8)	
H3A	−0.0579	0.5945	0.6301	0.048*	
H3B	−0.1896	0.6308	0.5552	0.048*	
C4	0.0174 (4)	0.68457 (14)	0.6044 (2)	0.0308 (7)	
C5	0.0024 (5)	0.73774 (17)	0.5430 (3)	0.0490 (9)	
H5	−0.0493	0.7354	0.4756	0.059*	
C6	0.0646 (6)	0.79525 (19)	0.5816 (4)	0.0696 (13)	
H6	0.0537	0.8314	0.5403	0.084*	
C7	0.1417 (6)	0.7982 (2)	0.6806 (5)	0.0775 (15)	
H7	0.1817	0.8367	0.7068	0.093*	
C8	0.1607 (6)	0.7456 (3)	0.7410 (4)	0.0752 (14)	
H8	0.2152	0.7479	0.8076	0.090*	
C9	0.0986 (5)	0.68877 (19)	0.7030 (3)	0.0517 (10)	
H9	0.1118	0.6528	0.7444	0.062*	
C10	0.3594 (4)	0.56283 (15)	0.0704 (2)	0.0385 (8)	
H10	0.4025	0.5627	0.1400	0.046*	
C11	0.3391 (4)	0.57667 (15)	−0.0972 (2)	0.0369 (7)	
H11	0.3652	0.5881	−0.1665	0.044*	
C12	0.6224 (7)	0.6171 (4)	−0.0050 (16)	0.040 (3)	0.68 (3)
H12A	0.7076	0.5936	−0.0462	0.048*	0.68 (3)
H12B	0.6633	0.6178	0.0684	0.048*	0.68 (3)
C13	0.608 (2)	0.6830 (4)	−0.0465 (10)	0.0356 (18)	0.68 (3)
C14	0.5342 (18)	0.7259 (6)	0.0216 (12)	0.049 (2)	0.68 (3)
H14	0.4990	0.7131	0.0887	0.058*	0.68 (3)
C15	0.5119 (14)	0.7881 (5)	−0.009 (2)	0.068 (4)	0.68 (3)
H15	0.4623	0.8171	0.0370	0.081*	0.68 (3)
C16	0.5640 (18)	0.8063 (5)	−0.1083 (18)	0.065 (5)	0.68 (3)
H16	0.5470	0.8478	−0.1295	0.078*	0.68 (3)
C17	0.639 (2)	0.7654 (8)	−0.1755 (12)	0.072 (4)	0.68 (3)
H17	0.6767	0.7787	−0.2417	0.086*	0.68 (3)
C18	0.660 (2)	0.7029 (7)	−0.1450 (11)	0.057 (3)	0.68 (3)
H18	0.7102	0.6743	−0.1917	0.069*	0.68 (3)
C12A	0.6197 (12)	0.6178 (8)	−0.026 (3)	0.034 (4)	0.32 (3)
H12C	0.6862	0.5951	−0.0794	0.041*	0.32 (3)
H12D	0.6862	0.6157	0.0400	0.041*	0.32 (3)
C13A	0.605 (4)	0.6853 (8)	−0.060 (2)	0.035 (4)	0.32 (3)
C14A	0.530 (3)	0.7370 (10)	−0.0096 (18)	0.038 (4)	0.32 (3)
H14A	0.4803	0.7324	0.0570	0.046*	0.32 (3)
C15A	0.530 (2)	0.7955 (8)	−0.059 (2)	0.048 (5)	0.32 (3)
H15A	0.4797	0.8300	−0.0254	0.057*	0.32 (3)
C16A	0.604 (2)	0.8023 (6)	−0.1587 (19)	0.047 (4)	0.32 (3)
H16A	0.6038	0.8415	−0.1917	0.056*	0.32 (3)
C17A	0.679 (3)	0.7507 (8)	−0.2089 (17)	0.044 (4)	0.32 (3)
H17A	0.7284	0.7553	−0.2755	0.053*	0.32 (3)
C18A	0.679 (4)	0.6922 (7)	−0.159 (2)	0.039 (5)	0.32 (3)
H18A	0.7289	0.6576	−0.1931	0.046*	0.32 (3)

### Atomic displacement parameters (Å^2^)

**Table:**

	*U*^11^	*U*^22^	*U*^33^	*U*^12^	*U*^13^	*U*^23^
Ag1	0.04650 (15)	0.04865 (17)	0.02972 (13)	−0.00637 (12)	−0.01010 (10)	−0.00375 (12)
V1	0.0375 (3)	0.0324 (3)	0.0249 (3)	0.0025 (2)	−0.0031 (2)	0.0009 (2)
F1	0.0848 (17)	0.121 (2)	0.0337 (12)	0.0688 (16)	−0.0171 (12)	−0.0150 (13)
F2	0.0453 (11)	0.0559 (13)	0.0398 (11)	−0.0063 (9)	−0.0038 (9)	0.0181 (9)
O1	0.0490 (14)	0.0397 (13)	0.0330 (12)	0.0045 (10)	−0.0085 (11)	−0.0072 (10)
O2	0.0326 (12)	0.0692 (16)	0.0253 (11)	−0.0118 (11)	−0.0045 (10)	0.0048 (11)
N1	0.0264 (13)	0.0414 (16)	0.0264 (13)	−0.0014 (11)	−0.0060 (11)	−0.0052 (11)
N2	0.0282 (13)	0.0329 (14)	0.0290 (14)	0.0001 (11)	−0.0083 (11)	−0.0044 (11)
N3	0.0341 (14)	0.0300 (14)	0.0235 (14)	−0.0013 (11)	−0.0001 (11)	−0.0039 (10)
N4	0.0502 (17)	0.0411 (16)	0.0242 (14)	−0.0115 (13)	−0.0049 (12)	0.0053 (12)
N5	0.0402 (15)	0.0408 (16)	0.0223 (13)	−0.0068 (12)	−0.0028 (12)	−0.0022 (11)
N6	0.0363 (14)	0.0373 (15)	0.0287 (14)	−0.0043 (11)	−0.0056 (12)	0.0021 (11)
C1	0.0256 (16)	0.0412 (19)	0.0341 (17)	0.0004 (13)	−0.0084 (14)	−0.0044 (14)
C2	0.0309 (16)	0.0373 (18)	0.0300 (17)	0.0005 (13)	−0.0085 (14)	−0.0051 (14)
C3	0.047 (2)	0.0395 (19)	0.0337 (18)	−0.0056 (15)	0.0107 (16)	−0.0084 (15)
C4	0.0270 (16)	0.0316 (17)	0.0339 (17)	0.0017 (13)	0.0031 (13)	−0.0065 (13)
C5	0.046 (2)	0.046 (2)	0.055 (2)	−0.0040 (17)	−0.0043 (18)	0.0073 (18)
C6	0.062 (3)	0.037 (2)	0.110 (4)	−0.004 (2)	0.013 (3)	0.009 (2)
C7	0.070 (3)	0.062 (3)	0.101 (4)	−0.024 (2)	0.023 (3)	−0.043 (3)
C8	0.067 (3)	0.105 (4)	0.053 (3)	−0.028 (3)	0.001 (2)	−0.034 (3)
C9	0.054 (2)	0.062 (3)	0.039 (2)	−0.0041 (19)	−0.0045 (18)	−0.0032 (18)
C10	0.052 (2)	0.0395 (19)	0.0239 (16)	−0.0072 (16)	−0.0098 (15)	0.0055 (14)
C11	0.0428 (19)	0.046 (2)	0.0220 (16)	−0.0028 (16)	−0.0040 (14)	0.0020 (14)
C12	0.036 (3)	0.049 (3)	0.036 (8)	−0.008 (3)	−0.010 (3)	0.006 (3)
C13	0.031 (3)	0.036 (3)	0.040 (4)	−0.008 (3)	−0.008 (3)	−0.004 (3)
C14	0.046 (4)	0.049 (5)	0.052 (5)	0.001 (4)	−0.003 (4)	−0.003 (4)
C15	0.046 (4)	0.042 (5)	0.115 (12)	0.002 (3)	−0.008 (6)	−0.012 (6)
C16	0.052 (6)	0.050 (5)	0.093 (13)	−0.016 (4)	−0.022 (7)	0.033 (6)
C17	0.080 (9)	0.081 (9)	0.053 (7)	−0.027 (7)	−0.009 (5)	0.019 (6)
C18	0.066 (8)	0.054 (5)	0.052 (6)	−0.017 (5)	−0.005 (5)	−0.008 (5)
C12A	0.037 (6)	0.046 (5)	0.020 (9)	0.001 (5)	−0.009 (4)	0.003 (4)
C13A	0.035 (6)	0.032 (5)	0.037 (7)	−0.004 (5)	−0.005 (6)	−0.001 (5)
C14A	0.034 (7)	0.044 (8)	0.036 (9)	−0.004 (5)	0.001 (7)	0.004 (6)
C15A	0.061 (11)	0.035 (8)	0.048 (11)	0.005 (7)	0.004 (9)	−0.001 (7)
C16A	0.045 (9)	0.040 (7)	0.055 (10)	−0.010 (6)	0.002 (7)	0.005 (7)
C17A	0.047 (9)	0.036 (7)	0.050 (9)	−0.006 (5)	0.005 (6)	0.000 (6)
C18A	0.048 (10)	0.036 (7)	0.032 (8)	−0.010 (6)	0.004 (6)	0.002 (5)

### Geometric parameters (Å, º)

**Table:**

Ag1—N5^i^	2.197 (2)	C7—H7	0.9300
Ag1—N1	2.233 (2)	C8—C9	1.378 (6)
Ag1—N4	2.390 (3)	C8—H8	0.9300
Ag1—O1	2.562 (2)	C9—H9	0.9300
V1—O1	1.632 (2)	C10—H10	0.9300
V1—O2	1.660 (2)	C11—H11	0.9300
V1—F1	1.828 (2)	C12—C13	1.497 (5)
V1—F2	1.8330 (18)	C12—H12A	0.9700
V1—N2	2.203 (2)	C12—H12B	0.9700
N1—C1	1.311 (4)	C13—C18	1.374 (9)
N1—N2	1.376 (3)	C13—C14	1.375 (8)
N2—C2	1.303 (4)	C14—C15	1.386 (11)
N3—C1	1.345 (4)	C14—H14	0.9300
N3—C2	1.345 (4)	C15—C16	1.371 (12)
N3—C3	1.473 (4)	C15—H15	0.9300
N4—C10	1.306 (4)	C16—C17	1.344 (13)
N4—N5	1.387 (3)	C16—H16	0.9300
N5—C11	1.306 (4)	C17—C18	1.390 (11)
N5—Ag1^i^	2.197 (2)	C17—H17	0.9300
N6—C10	1.339 (4)	C18—H18	0.9300
N6—C11	1.344 (4)	C12A—C13A	1.497 (6)
N6—C12A	1.484 (5)	C12A—H12C	0.9700
N6—C12	1.484 (5)	C12A—H12D	0.9700
C1—H1	0.9300	C13A—C14A	1.3900
C2—H2	0.9300	C13A—C18A	1.3900
C3—C4	1.497 (4)	C14A—C15A	1.3900
C3—H3A	0.9700	C14A—H14A	0.9300
C3—H3B	0.9700	C15A—C16A	1.3900
C4—C5	1.372 (5)	C15A—H15A	0.9300
C4—C9	1.378 (4)	C16A—C17A	1.3900
C5—C6	1.393 (5)	C16A—H16A	0.9300
C5—H5	0.9300	C17A—C18A	1.3900
C6—C7	1.369 (6)	C17A—H17A	0.9300
C6—H6	0.9300	C18A—H18A	0.9300
C7—C8	1.358 (7)		
			
N5^i^—Ag1—N1	140.62 (9)	C7—C8—C9	119.6 (4)
N5^i^—Ag1—N4	102.45 (9)	C7—C8—H8	120.2
N1—Ag1—N4	112.90 (9)	C9—C8—H8	120.2
N5^i^—Ag1—O1	129.87 (8)	C8—C9—C4	120.9 (4)
N1—Ag1—O1	75.28 (8)	C8—C9—H9	119.6
N4—Ag1—O1	79.39 (8)	C4—C9—H9	119.6
O1—V1—O2	108.04 (11)	N4—C10—N6	110.8 (3)
O1—V1—F1	99.57 (11)	N4—C10—H10	124.6
O2—V1—F1	99.21 (13)	N6—C10—H10	124.6
O1—V1—F2	117.63 (10)	N5—C11—N6	110.5 (3)
O2—V1—F2	132.25 (10)	N5—C11—H11	124.8
F1—V1—F2	86.76 (10)	N6—C11—H11	124.8
O1—V1—N2	87.14 (10)	N6—C12—C13	109.3 (8)
O2—V1—N2	88.78 (11)	N6—C12—H12A	109.8
F1—V1—N2	167.32 (10)	C13—C12—H12A	109.8
F2—V1—N2	80.59 (9)	N6—C12—H12B	109.8
V1—O1—Ag1	128.89 (11)	C13—C12—H12B	109.8
C1—N1—N2	106.4 (2)	H12A—C12—H12B	108.3
C1—N1—Ag1	132.19 (19)	C18—C13—C14	118.9 (7)
N2—N1—Ag1	121.35 (18)	C18—C13—C12	125.6 (13)
C2—N2—N1	107.3 (2)	C14—C13—C12	115.5 (13)
C2—N2—V1	127.4 (2)	C13—C14—C15	120.4 (8)
N1—N2—V1	124.36 (18)	C13—C14—H14	119.8
C1—N3—C2	105.0 (2)	C15—C14—H14	119.8
C1—N3—C3	127.7 (3)	C16—C15—C14	119.2 (9)
C2—N3—C3	126.7 (3)	C16—C15—H15	120.4
C10—N4—N5	106.5 (2)	C14—C15—H15	120.4
C10—N4—Ag1	120.2 (2)	C17—C16—C15	121.4 (8)
N5—N4—Ag1	133.30 (19)	C17—C16—H16	119.3
C11—N5—N4	106.8 (2)	C15—C16—H16	119.3
C11—N5—Ag1^i^	128.7 (2)	C16—C17—C18	119.4 (8)
N4—N5—Ag1^i^	122.94 (19)	C16—C17—H17	120.3
C10—N6—C11	105.4 (3)	C18—C17—H17	120.3
C10—N6—C12A	134.8 (15)	C13—C18—C17	120.7 (9)
C11—N6—C12A	119.7 (15)	C13—C18—H18	119.7
C10—N6—C12	124.3 (8)	C17—C18—H18	119.7
C11—N6—C12	130.2 (7)	N6—C12A—C13A	113.8 (16)
N1—C1—N3	110.7 (3)	N6—C12A—H12C	108.8
N1—C1—H1	124.6	C13A—C12A—H12C	108.8
N3—C1—H1	124.6	N6—C12A—H12D	108.8
N2—C2—N3	110.6 (3)	C13A—C12A—H12D	108.8
N2—C2—H2	124.7	H12C—C12A—H12D	107.7
N3—C2—H2	124.7	C14A—C13A—C18A	120.0
N3—C3—C4	115.5 (3)	C14A—C13A—C12A	131.1 (18)
N3—C3—H3A	108.4	C18A—C13A—C12A	108.9 (19)
C4—C3—H3A	108.4	C13A—C14A—C15A	120.0
N3—C3—H3B	108.4	C13A—C14A—H14A	120.0
C4—C3—H3B	108.4	C15A—C14A—H14A	120.0
H3A—C3—H3B	107.5	C16A—C15A—C14A	120.0
C5—C4—C9	119.0 (3)	C16A—C15A—H15A	120.0
C5—C4—C3	121.6 (3)	C14A—C15A—H15A	120.0
C9—C4—C3	119.2 (3)	C15A—C16A—C17A	120.0
C4—C5—C6	120.1 (4)	C15A—C16A—H16A	120.0
C4—C5—H5	120.0	C17A—C16A—H16A	120.0
C6—C5—H5	120.0	C18A—C17A—C16A	120.0
C7—C6—C5	119.6 (4)	C18A—C17A—H17A	120.0
C7—C6—H6	120.2	C16A—C17A—H17A	120.0
C5—C6—H6	120.2	C17A—C18A—C13A	120.0
C8—C7—C6	120.8 (4)	C17A—C18A—H18A	120.0
C8—C7—H7	119.6	C13A—C18A—H18A	120.0
C6—C7—H7	119.6		
			
O2—V1—O1—Ag1	−74.41 (17)	Ag1—N4—C10—N6	−178.0 (2)
F1—V1—O1—Ag1	−177.46 (15)	C11—N6—C10—N4	−0.4 (4)
F2—V1—O1—Ag1	91.19 (15)	C12A—N6—C10—N4	178.8 (12)
N2—V1—O1—Ag1	13.37 (14)	C12—N6—C10—N4	178.0 (6)
C1—N1—N2—C2	−0.7 (3)	N4—N5—C11—N6	0.1 (4)
Ag1—N1—N2—C2	177.7 (2)	Ag1^i^—N5—C11—N6	−165.5 (2)
C1—N1—N2—V1	168.9 (2)	C10—N6—C11—N5	0.2 (4)
Ag1—N1—N2—V1	−12.7 (3)	C12A—N6—C11—N5	−179.2 (10)
C10—N4—N5—C11	−0.4 (4)	C12—N6—C11—N5	−178.1 (7)
Ag1—N4—N5—C11	177.9 (2)	C10—N6—C12—C13	−120.7 (10)
C10—N4—N5—Ag1^i^	166.2 (2)	C11—N6—C12—C13	57.3 (15)
Ag1—N4—N5—Ag1^i^	−15.5 (4)	N6—C12—C13—C18	−101.7 (13)
N2—N1—C1—N3	1.3 (3)	N6—C12—C13—C14	77.8 (15)
Ag1—N1—C1—N3	−176.8 (2)	C18—C13—C14—C15	0.4 (10)
C2—N3—C1—N1	−1.4 (3)	C12—C13—C14—C15	−179.1 (13)
C3—N3—C1—N1	−172.8 (3)	C13—C14—C15—C16	0.1 (13)
N1—N2—C2—N3	−0.2 (3)	C14—C15—C16—C17	−1.3 (15)
V1—N2—C2—N3	−169.37 (19)	C15—C16—C17—C18	1.8 (15)
C1—N3—C2—N2	1.0 (3)	C14—C13—C18—C17	0.0 (10)
C3—N3—C2—N2	172.5 (3)	C12—C13—C18—C17	179.5 (15)
C1—N3—C3—C4	−128.2 (3)	C16—C17—C18—C13	−1.1 (13)
C2—N3—C3—C4	62.2 (4)	C10—N6—C12A—C13A	−113 (2)
N3—C3—C4—C5	68.5 (4)	C11—N6—C12A—C13A	66 (3)
N3—C3—C4—C9	−116.6 (3)	N6—C12A—C13A—C14A	62 (4)
C9—C4—C5—C6	−1.7 (5)	N6—C12A—C13A—C18A	−117 (2)
C3—C4—C5—C6	173.3 (3)	C18A—C13A—C14A—C15A	0.0
C4—C5—C6—C7	0.5 (6)	C12A—C13A—C14A—C15A	−179 (3)
C5—C6—C7—C8	0.9 (7)	C13A—C14A—C15A—C16A	0.0
C6—C7—C8—C9	−1.1 (7)	C14A—C15A—C16A—C17A	0.0
C7—C8—C9—C4	−0.1 (7)	C15A—C16A—C17A—C18A	0.0
C5—C4—C9—C8	1.5 (5)	C16A—C17A—C18A—C13A	0.0
C3—C4—C9—C8	−173.6 (4)	C14A—C13A—C18A—C17A	0.0
N5—N4—C10—N6	0.5 (4)	C12A—C13A—C18A—C17A	179 (2)

Symmetry code: (i) −*x*, −*y*+1, −*z*.

### Hydrogen-bond geometry (Å, º)

**Table:**

*D*—H···*A*	*D*—H	H···*A*	*D*···*A*	*D*—H···*A*
C1—H1···O2^ii^	0.93	2.44	3.289 (4)	153
C1—H1···F2^iii^	0.93	2.63	3.108 (4)	113
C2—H2···F1^iv^	0.93	2.07	2.935 (4)	154
C2—H2···F2^iv^	0.93	2.60	3.304 (4)	133
C3—H3*A*···O1^iii^	0.97	2.73	3.465 (4)	133
C3—H3*B*···F2^iii^	0.97	2.37	3.006 (4)	123
C10—H10···O2	0.93	2.16	3.082 (4)	170
C11—H11···F1^v^	0.93	2.07	2.935 (4)	153
C12—H12*A*···O1^v^	0.97	2.65	3.388 (2)	133
C16—H16···O2^vi^	0.93	2.42	3.339 (9)	172
C18—H18···O1^v^	0.93	2.83	3.589 (15)	139

Symmetry codes: (ii) *x*−1, *y*, *z*; (iii) −*x*, −*y*+1, −*z*+1; (iv) −*x*+1, −*y*+1, −*z*+1; (v) −*x*+1, −*y*+1, −*z*; (vi) *x*, −*y*+3/2, *z*−1/2.
